# Tau-PET and in vivo Braak-staging as prognostic markers of future cognitive decline in cognitively normal to demented individuals

**DOI:** 10.1186/s13195-021-00880-x

**Published:** 2021-08-12

**Authors:** Davina Biel, Matthias Brendel, Anna Rubinski, Katharina Buerger, Daniel Janowitz, Martin Dichgans, Nicolai Franzmeier, for the Alzheimer’s Disease Neuroimaging Initiative (ADNI)

**Affiliations:** 1 Institute, for Stroke and Dementia Research (ISD), University Hospital, LMU Munich, 81377 Munich, Germany; 2 Department of Nuclear Medicine, University Hospital, LMU Munich, 80336 Munich, Germany; 3 German Center for Neurodegenerative Diseases (DZNE, Munich), Munich, Germany; 4 Munich Cluster for Systems Neurology (SyNergy), Munich, Germany

**Keywords:** Alzheimer’s disease, Braak-staging, Tau-PET, Amyloid-PET, Conversion risk

## Abstract

**Background:**

To systematically examine the clinical utility of tau-PET and Braak-staging as prognostic markers of future cognitive decline in older adults with and without cognitive impairment.

**Methods:**

In this longitudinal study, we included 396 cognitively normal to dementia subjects with ^18^F-Florbetapir/^18^F-Florbetaben-amyloid-PET, ^18^F-Flortaucipir-tau-PET and ~ 2-year cognitive follow-up. Annual change rates in global cognition (i.e., MMSE, ADAS13) and episodic memory were calculated via linear-mixed models. We determined global amyloid-PET (Centiloid) plus global and Braak-stage-specific tau-PET SUVRs, which were stratified as positive(^+^)/negative(^−^) at pre-established cut-offs, classifying subjects as Braak^0^/Braak^I+^/Braak^I–IV+^/Braak^I–VI+^/Braak^atypical+^. In bootstrapped linear regression, we assessed the predictive accuracy of global tau-PET SUVRs vs. Centiloid on subsequent cognitive decline. To test for independent tau vs. amyloid effects, analyses were further controlled for the contrary PET-tracer. Using ANCOVAs, we tested whether more advanced Braak-stage predicted accelerated future cognitive decline. All models were controlled for age, sex, education, diagnosis, and baseline cognition. Lastly, we determined Braak-stage-specific conversion risk to mild cognitive impairment (MCI) or dementia.

**Results:**

Baseline global tau-PET SUVRs explained more variance (partial *R*^2^) in future cognitive decline than Centiloid across all cognitive tests (Cohen’s *d* ~ 2, all tests *p* < 0.001) and diagnostic groups. Associations between tau-PET and cognitive decline remained consistent when controlling for Centiloid, while associations between amyloid-PET and cognitive decline were non-significant when controlling for tau-PET. More advanced Braak-stage was associated with gradually worsening future cognitive decline, independent of Centiloid or diagnostic group (*p* < 0.001), and elevated conversion risk to MCI/dementia.

**Conclusion:**

Tau-PET and Braak-staging are highly predictive markers of future cognitive decline and may be promising single-modality estimates for prognostication of patient-specific progression risk in clinical settings.

**Supplementary Information:**

The online version contains supplementary material available at 10.1186/s13195-021-00880-x.

## Background

Beta-amyloid (Aβ) and tau are hallmark pathologies of Alzheimer’s disease (AD), ensuing neurodegeneration, cognitive decline, and dementia [1, 2]. The development of in vivo Aβ and tau biomarkers has greatly facilitated diagnosing AD [2]; however, a reliable prognosis of AD-related cognitive decline in clinical settings remains a critical yet unmet challenge. Notably, tau pathology emerges much closer to symptom onset than Aβ in AD [1, 2], as revealed by positron-emission-tomography (PET) [3, 4], biofluid biomarkers [5], and post-mortem examinations [6, 7]. Moreover, PET-based tau assessments show a strong association with cross-sectional cognition [8] and cognitive decline [9–12]. In contrast to spatially diffuse Aβ accumulation [13], tau typically spreads systematically across the temporal lobe, association cortices, and eventually primary sensorimotor cortices in amnestic/typical AD, as summarized in the Braak-staging scheme of progressing tau pathology [14, 15]. This spatio-temporal progression of tau has been closely related to disease stage and cognitive performance [14, 15]. Thus, tau-PET and Braak-staging might facilitate patient-specific risk estimation of future cognitive decline in clinical settings, which would have implications for clinical decision making (e.g., interventions/intensified care) or risk matching of subjects in clinical trials. To systematically investigate tau-PET as a single predictive marker for future cognitive decline, we included 396 subjects from the Alzheimer’s disease neuroimaging initiative (ADNI) ranging from cognitively normal to AD dementia, characterized by baseline ^18^F-Florbetapir/^18^F-Florbetaben amyloid-PET, ^18^F-Flortaucipir tau-PET, and ~ 2-year follow-up assessments of global cognitive and memory performance. We tested (i) the predictive accuracy of tau-PET for future cognitive decline vs. amyloid-PET and assessed (ii) whether tau-PET-based Braak-staging facilitates gradual prediction of future cognitive worsening and clinical AD progression.

## Methods

### Participants

**Fig. 1:**
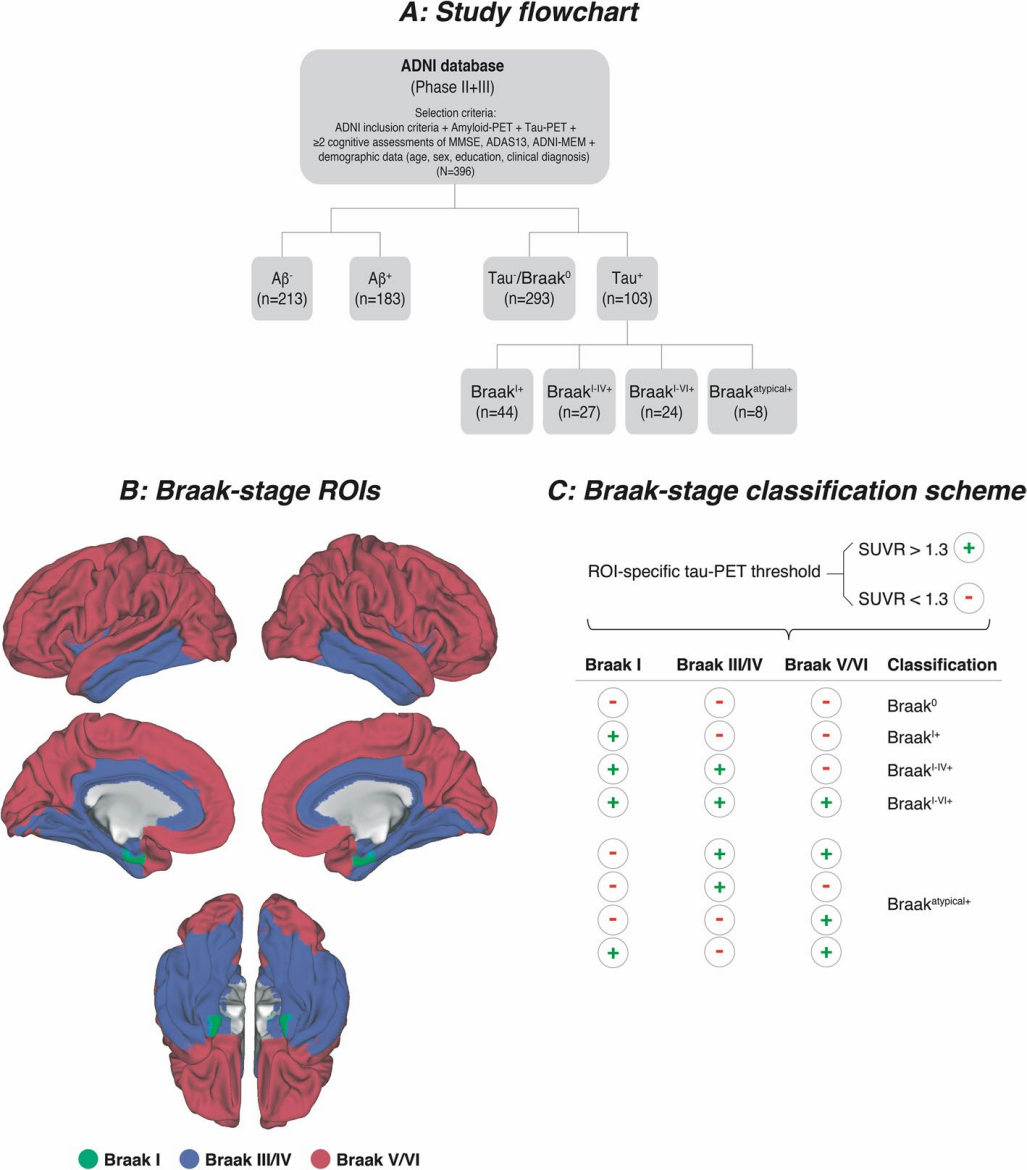
Study flowchart (**A**), surface rendering of Braak-stage ROIs applied to tau-PET data (**B**), and tau-PET classification of subjects into Braak-stages (**C**)

We included 396 participants from the ADNI database. Beyond ADNI inclusion criteria (https://adni.loni.usc.edu/wp-content/uploads/2010/09/ADNI_GeneralProceduresManual.pdf), the current study required availability of amyloid-PET (either ^18^F-Florbetapir-PET, *n* = 280, or ^18^F-Florbetaben-PET, *n* = 116), together with ^18^F-Flortaucipir tau-PET and longitudinal cognitive assessments (≥ 2 examinations), demographics (age, sex, education) and clinical diagnosis. Subjects were categorized by ADNI as cognitively normal (CN, Mini-Mental State Examination [MMSE] ≥ 24, Clinical Dementia Rating [CDR] = 0, non-depressed), mildly cognitively impaired (MCI, MMSE ≥ 24, CDR = 0.5, objective memory-impairment on education adjusted Wechsler Memory Scale II, preserved activities of daily living) or demented (MMSE = 20–26, CDR > 0.5, NINCDS/ADRDA criteria for probable AD). For the current study, all baseline PET and cognitive data had to be obtained within a time-window of 6 months. A study flowchart is displayed in Fig. 1A.

### Assessment of cognitive changes

We included longitudinal measures of global cognition and memory. For global cognition, we used the MMSE, i.e., a screening instrument for cognitive deficits that is widely used in clinical routine [16], as well as the more extensive Alzheimer’s Disease Assessment Scale Cognition 13-item scale (ADAS13) [17]. Memory performance was assessed using the pre-established ADNI-MEM composite score [18], which includes the Rey Auditory Verbal Learning Test, the Alzheimer’s Disease Assessment Scale, the Wechsler Logical Memory Scale I and II, and MMSE word recall. The average cognitive follow-up time was 2.02 years (range = 1.00–4.50 years, mean = 2.53 cognitive assessments). To determine annual cognitive change rates, we used a pre-established approach [19, 20], fitting linear mixed models with MMSE, ADAS13, and ADNI-MEM scores as the dependent variable and time (i.e., years from baseline) as the independent variable, controlling for random slope and intercept. From the linear mixed models, we then derived a slope estimate for change in MMSE, ADAS13, and ADNI-MEM across time (i.e., change per year) for each subject.

### Neuroimaging acquisition and PET preprocessing

3 T Structural MRI was obtained via T1-weighted MPRAGE sequences using unified scanning protocols (sequence details can be found on: http://adni.loni.usc.edu/methods/mri-tool/mri-analysis/). Amyloid-PET was acquired 50–70 min after ^18^F-Florbetapir injection in 4 × 5 min frames or 90–110 min after ^18^F-Florbetaben injection in 4 × 5 min frames. Tau-PET was acquired 75–105 min after injection of ^18^F-Flortaucipir in 6 × 5 min frames. For each tracer, recorded timeframes were motion corrected and averaged to obtain a mean image (see also http://adni.loni.usc.edu/methods/pet-analysis-method/pet-analysis/). Structural MRI was preprocessed by ADNI using standard Freesurfer pipelines.

For amyloid-PET, the MRI-derived Freesurfer parcellation [21] was applied to co-registered PET images to extract global amyloid-PET standardized uptake value ratio (SUVR) values intensity normalized to the whole cerebellum as described previously (see also https://adni.bitbucket.io/reference/docs/UCBERKELEYAV45/ADNI_AV45_Methods_JagustLab_06.25.15.pdf and https://adni.bitbucket.io/reference/docs/UCBERKELEYFBB/UCBerkeley_FBB_Methods_04.11.19.pdf) [22–26]. Subjects were classified as Aβ^+^ when surpassing pre-established SUVR thresholds (^18^F-Florbetapir SUVR > 1.11 [25]; ^18^F-Florbetaben SUVR > 1.08, Fig. 1A). To harmonize global amyloid-PET SUVRs across ^18^F-Florbetapir and ^18^F-Florbetaben tracers, global SUVR values were transformed to Centiloid (CL) using equations provided by ADNI [27]. For tau-PET, images were co-registered to structural MRI to extract mean Freesurfer ROI values which were SUVR normalized to the inferior cerebellar gray, following a pre-established approach [28]. For tau-PET, we obtained global and Braak-stage-ROI-specific (Fig. 1B) tau-PET SUVR scores. For global tau, we averaged SUVRs across cortical Freesurfer ROIs, excluding the cerebellum, hippocampus, thalamus, and basal ganglia (i.e., typical regions of ^18^F-Flortaucipir off-target binding) following a previously described approach [28]. For Braak-stage specific tau-PET, we applied in vivo Braak-staging that allows application of the post-mortem established Braak tau staging system to tau-PET imaging (see Fig. 1B) [15]. A list of Freesurfer ROIs included within each Braak-stage ROI can be found online (https://adni.bitbucket.io/reference/docs/UCBERKELEYAV1451/UCBERKELEY_AV1451_Methods_Aug2018.pdf). In brief, we obtained tau-PET SUVRs for Braak-stage I, Braak-stage III/IV, and Braak-stage V/VI composite ROIs. Braak-stage II (i.e., hippocampus) was excluded due to ^18^F-Flortaucipir off-target binding in this region [29].

### Braak-staging

Subjects were defined as tau^+^ when at least one Braak-stage ROI surpassed a pre-established cut-off of 1.3 SUVR [23, 28]. For Braak-staging, subjects were classified as Braak I positive (i.e., Braak^I+^, *n* = 44), when only the Braak I ROI (i.e., entorhinal cortex) surpassed a SUVR cut-off of 1.3. Subjects were classified as Braak I–IV positive (i.e., Braak^I–IV+^, *n* = 27) when both Braak I and Braak III/IV ROIs surpassed the SUVR cut-off of 1.3, and as Braak I–VI positive (i.e., Braak^I–VI+^, *n* = 24), when Braak ROIs I, III/IV and V/VI surpassed the 1.3 SUVR threshold. Subjects who deviated from this staging scheme (e.g., for which Braak I was negative, but Braak III/IV was positive) were labeled Braak atypical (i.e., Braak^atypical+^, *n* = 8). Subjects were classified as Braak^0^/tau^−^ (*n* = 293), when all Braak ROIs had an SUVR below 1.3 (Fig. 1A). The Braak-staging scheme is illustrated in Fig. 1C. The rationale of restricting our Braak-staging analysis to a single cut-off SUVR of 1.3 in contrast to previous studies (e.g., using Braak-stage specific cut-offs) [28] was to implement an approach that can be easily and uniformly applied in clinical settings. Note that exploratory altering the tau-PET threshold between 1.2–1.4 yielded congruent results with those presented in the manuscript.

### Statistical analysis

Differences in baseline characteristics between diagnostic groups were assessed using ANOVAs for continuous and chi-squared (*χ*^2^) tests for categorial data. To test tau-PET and amyloid-PET as predictors of longitudinal cognitive change rates, we performed linear regression, using annual cognitive change rates (i.e., MMSE, ADAS13, and ADNI-MEM), as dependent variables, and baseline PET (global tau-PET SUVR vs. global amyloid-PET SUVR transformed to CL) as independent variables. Regression models were controlled for age, sex, education, diagnosis, and the baseline score of the respective cognitive test (i.e., MMSE, ADAS13, or ADNI-MEM). To determine the variance that tau-PET or amyloid-PET explained in longitudinal cognitive changes, we calculated partial R^2^ values for either tau- or amyloid-PET as predictors of cognitive changes. In order to assess the accuracy of tau- and amyloid-PET in predicting future cognitive decline, we performed bootstrapping, repeating the above described regression models on 1000 bootstrapped samples and compared the resulting partial *R*^2^ values using paired t-tests. Standardized differences between partial *R*^2^ distributions of tau-PET and amyloid-PET were calculated using Cohen's *d*. For non-parametric comparison, we further determined 95% confidence intervals (CI) of bootstrapped partial *R*^2^ distributions of tau-PET and amyloid-PET as predictors of cognitive changes. Subsequently, regression models were repeated, this time controlling for global amyloid-PET when assessing global tau-PET as a predictor, and controlling for global tau-PET when assessing global amyloid-PET as a predictor of cognitive decline. The rationale was to ensure that the specific predictor which was assessed in the regression model (i.e., tau-PET or amyloid-PET) explained additional variance in cognitive decline. Further, we exploratory assessed whether the associations between tau- and amyloid-PET and cognition were consistent across diagnostic groups, i.e., we repeated the above described analyses stratified by diagnostic groups (CN, MCI, dementia). Next, we tested whether more advanced Braak-stage was associated with increased risk of future cognitive decline. To this end, we ran ANCOVAs, using annual cognitive change rates as dependent variables (i.e., MMSE, ADAS13, and ADNI-MEM) and Braak-stage (i.e., Braak^0^/Braak^I+^/Braak^I–IV+^/Braak^I–VI+^/Braak^atypical+^) as independent variable, controlling for age, sex, education, diagnosis, global amyloid-PET, and the baseline score of the respective cognitive test. Standardized differences in cognitive decline between Braak-stage groups were determined using Cohen’s *d*. Again, we repeated the above described analyses stratified by diagnostic group, to exploratory assess whether Braak-stage specific associations with cognitive decline were consistent across clinical stages. Lastly, we determined for each Braak-stage group the risk of clinical conversion, defined as the relative risk of a change in diagnosis from CN to MCI/dementia or from MCI to dementia during follow-up. Subjects with a baseline diagnosis of dementia were excluded from this analysis, since no further conversion can be diagnosed in these patients. Conversion rates were compared using *χ*^2^ tests.

All analyses were computed using R statistical software version 4.0.2 (r-project.org [30]). For primary analyses on cognitive measures (MMSE, ADAS13, ADNI-MEM), Bonferroni correction was applied (adjusted alpha level: 0.05/3 = 0.017). Post hoc Bonferroni-corrected Tukey tests were applied to ANCOVAs.

## Results

The sample included 239 CN, 122 MCI, and 35 demented individuals. From the CN group, 14 converted to MCI during one of the follow-ups (i.e., 5.86% conversion rate), with no conversions to dementia observed. In MCI, 14 converted to dementia during one of the follow-ups (i.e., 11.48% conversion rate). Descriptive statistics are displayed in Table 1.

**Table 1:** Subjects characteristics

**Amyloid**^**+**^** (*n* = 183)**	**CN (*n* = 93)**	**MCI (*n* = 60)**	**Dementia (*n* = 30)**	***p* value**
Age in years	75.67 (7.25)	76.16 (6.82)	76.95 (7.90)	0.692
Sex (male/female)	40/53	35/25	14/16	0.175
Years of education	16.59 (2.30)	15.78 (2.73)	16.1 (2.34)	0.132
MMSE score	28.83 (1.48)^b,c^	27.2 (2.32)^a,c^	22.27 (3.85)^a,b^	< 0.001
ADAS13 score	12.88 (5.27)^b,c^	19.44 (6.92)^a,c^	32.50 (9.32)^a,b^	< 0.001
ADNI-MEM score	0.95 (0.56)^b,c^	0.13 (0.60)^a,c^	− 0.73 (0.73)^a,b^	< 0.001
Amyloid-PET CL	62.10 (32.54)^c^	75.72 (37.44)	88.30 (36.61)^a^	< 0.001
Global Tau-PET SUVR	1.13 (0.09)^b,c^	1.22 (0.18)^a,c^	1.38 (0.36)^a,b^	< 0.001
Braak^0^/Braak^I+^/Braak^I–IV+^/Braak^I–VI+^/Braak^atypical+^	68/16/6/1/2	22/14/13/8/3	6/4/6/13/1	< 0.001
Mean cognitive follow-up in years	2.03 (0.68)	1.88 (0.87)	1.67 (0.73)	0.072
**Amyloid**^**−**^** (*n* = 213)**	**CN (*n* = 146)**	**MCI (*n* = 62)**	**Dementia (*n* = 5)**	***p* value**
Age in years	72.76 (6.87) ^b^	75.47 (8.60) ^a^	71.22 (7.45)	0.045
Sex (female/male)	60/86	39/23	3/2	0.014
Years of education	16.95 (2.42)^c^	16.18 (3.00)	14 (2.83)^a^	0.011
MMSE score	29.21 (0.98)^b,c^	28.56 (1.72)^a,c^	24.4 (1.14)^a,b^	< 0.001
ADAS13 score	11.16 (4.29)^b,c^	16.19 (5.43)^a,c^	30.07 (4.39)^a,b^	< 0.001
ADNI-MEM score	1.10 (0.60)^b,c^	0.60 (0.56)^a,c^	− 0.61 (0.59)^a,b^	< 0.001
Amyloid-PET CL	5.60 (8.82)^b^	− 0.72 (12.34)^a^	− 3.03 (8.87)	< 0.001
Global Tau-PET SUVR	1.09 (0.07)	1.08 (0.08)	1.08 (0.07)	0.811
Braak^0^/Braak^I+^/Braak^I–IV+^/Braak^I–VI+^/Braak^atypical+^	137/5/1/2/1	56/4/1/0/1	4/1/0/0/0	0.718
Mean cognitive follow-up in years	2.18 (0.62)	2.01 (0.92)	1.53 (0.58)	0.056

Values are presented as mean (SD)

*p* values were derived from ANOVAs for continuous measures and from chi-squared tests for categorical measures

*MMSE* Mini-Mental State Examination, *ADAS13* Alzheimer’s disease assessment scale, cognitive subscale, *ADNI-MEM* episodic memory composite score

Mean values significantly (*p* < 0.05, post hoc tests) different from ^a^CN, ^b^MCI, and ^c^Dementia

### Global tau-PET is a better predictor of future cognitive decline than global amyloid-PET

**Fig. 2:**
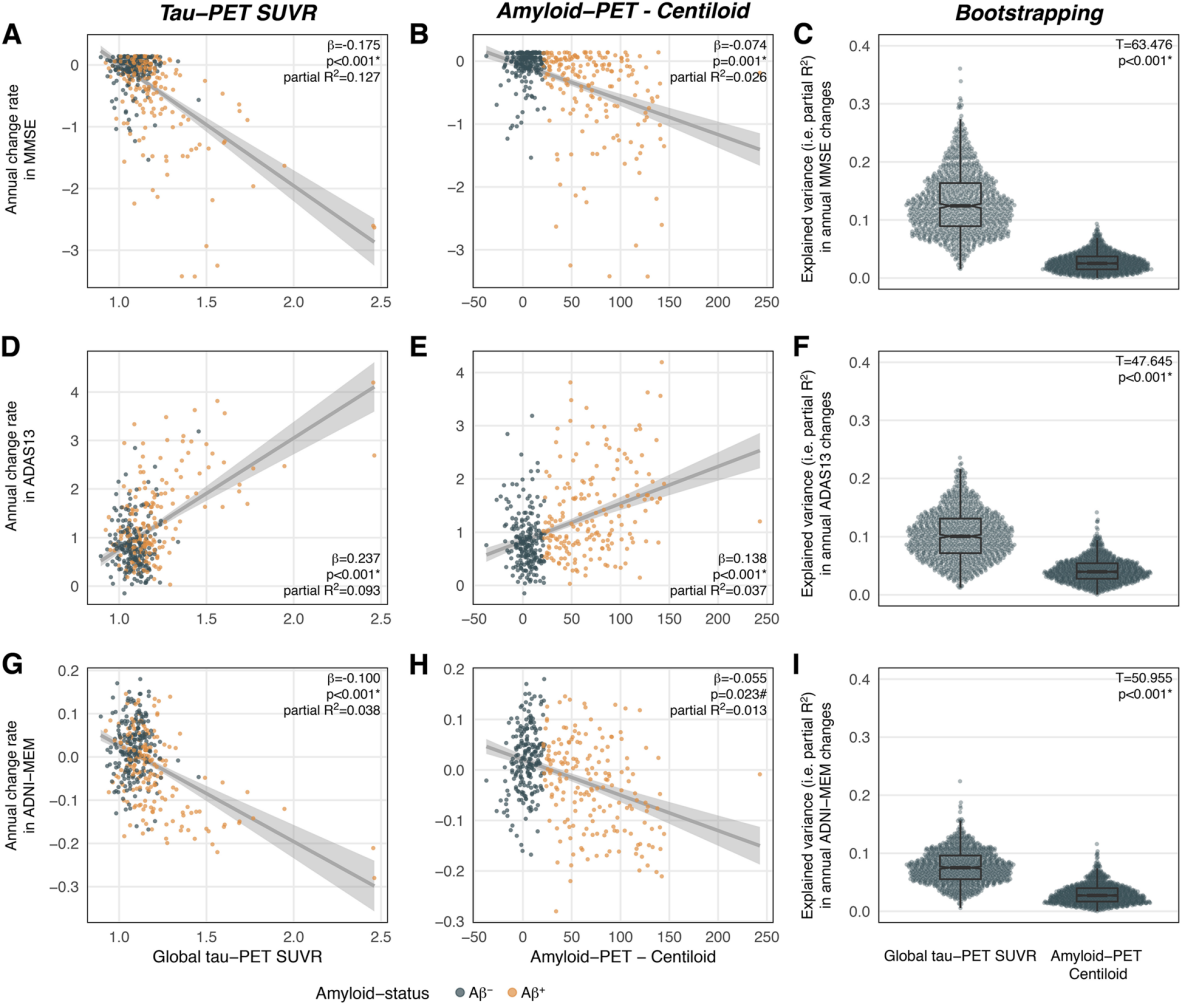
Scatterplot illustrating the association between global tau-PET SUVRs, baseline global amyloid-PET (i.e., Centiloid), and annual cognitive changes of the Mini Mental State Examination (MMSE; **A** + **B**), Alzheimer’s Disease Assessment Scale Cognition 13-item scale (ADAS13; **D** + **E**), and ADNI-MEM (**G** + **H**). Standardized beta-values were derived from linear regression controlling for age, sex, education, clinical diagnosis, and the baseline score of the respective cognitive test. Bootstrapping analysis with 1000 iterations (**C** + **F** + **I**) revealed that bootstrapped distributions of partial *R*^2^ values (i.e., explained variance in cognitive changes) were higher for global tau-PET than for global amyloid-PET. Bonferroni correction applied, adjusted alpha level = 0.017; significant p-values are marked with *; uncorrected significant *p* values (*p* < 0.05) are marked with #. Note, that the association between amyloid-PET and ADNI-MEM did not survive Bonferroni correction

First, we tested the predictive accuracy of global tau-PET for future cognitive decline compared to global amyloid-PET (i.e., CL). In linear regression, higher baseline global tau-PET SUVRs were associated with faster cognitive changes (MMSE: β =  − 0.175, *T* =  − 7.528, *p* < 0.001, partial *R*^2^ = 0.127, Fig. 2A; ADAS13: *β* = 0.237, *T* = 6.329, *p* < 0.001, partial *R*^2^ = 0.093, Fig. 2D; ADNI-MEM: *β* =  − 0.100, *T* =  − 3.927, *p* < 0.001, partial *R*^2^ = 0.038, Fig. 2G). Similarly, higher amyloid-PET was associated with faster subsequent longitudinal cognitive decline for the MMSE (*β* =  − 0.074, *T* =  − 3.205, *p* = 0.001, partial *R*^2^ = 0.026, Fig. 2B) and ADAS13 (*β* = 0.138, *T* = 3.830, *p* < 0.001, partial *R*^2^ = 0.037, Fig. 2E), while results for ADNI-MEM did not survive Bonferroni correction (*p* = 0.023, Fig. 2H). Notably, the variance explained (i.e., partial *R*^2^) in cognitive decline was higher for global tau-PET compared to global amyloid-PET. Repeating the above decribed analyses using robust regression yielded congruent results, suggesting that our results were not particularly driven by extreme values or skewed data. To statistically compare the predictive accuracy of tau-PET vs. amyloid-PET, we performed bootstrapping. Confirming a higher accuracy of tau-PET vs. amyloid-PET for predicting future cognitive decline, bootstrapped distributions of partial *R*^2^ values were higher for global tau-PET than for global amyloid-PET (MMSE: *T* = 63.476, *p* < 0.001, Cohen’s *d* = 2.38, Fig. 2C; ADAS13: *T* = 47.645, *p* < 0.001, Cohen’s *d* = 1.76, Fig. 2F; ADNI-MEM: *T* = 50.955, *p* < 0.001, Cohen’s *d* = 1.94, Fig. 2I). In addition, the 95%CIs of the bootstrapped partial *R*^2^ distributions did not overlap for MMSE (amyloid-PET: CI = 0.026–0.028, mean = 0.027; tau-PET: CI = 0.126–0.133, mean = 0.130), ADAS13 (amyloid-PET: CI = 0.041–0.043, mean = 0.042; tau-PET: CI = 0.100–0.105, mean = 0.103), or ADNI-MEM (amyloid-PET: CI = 0.029–0.031, mean = 0.030; tau-PET: CI = 0.075–0.078, mean = 0.077), providing non-parametric support of a significant difference between amyloid-PET and tau-PET-derived partial *R*^2^ distributions.

In a next step, we assessed whether the observed associations between tau-PET and cognition were independent of amyloid-PET and vice versa. To this end, the regression models for tau- and amyloid-PET were repeated, this time additionally controlling for the other respective PET-tracer. Importantly, all associations between tau-PET and cognitive decline remained consistent when additionally controlling for global amyloid-PET (MMSE: *β* =  − 0.167, *T* =  − 6.791, *p* < 0.001, partial *R*^2^ for tau-PET = 0.106, partial *R*^2^ for amyloid-PET = 0.002; ADAS13: *β* = 0.211, *T* = 5.371, *p* < 0.001, partial *R*^2^ for tau-PET = 0.069, partial *R*^2^ for amyloid-PET = 0.011; ADNI-MEM: *β* =  − 0.091, *T* =  − 3.354, *p* < 0.001, partial *R*^2^ for tau-PET = 0.028, partial *R*^2^ for amyloid-PET = 0.003). In contrast, no associations between amyloid-PET and cognitive decline reached statistical significance when additionally controlling for tau-PET. This finding supports tau-PET as a much better predictor for cognitive decline than amyloid-PET when it comes to prognostication of future cognitive decline. To test whether this result pattern is consistent across diagnostic groups, the regression analyses were repeated stratified by diagnostic group (i.e., CN, MCI, dementia), controlling for age, sex, education, baseline cognition, and the contrary PET-tracer. We found significant associations between global tau-PET SUVRs and cognitive decline across diagnostic groups within CN (MMSE: *β* =  − 0.100, *T* =  − 2.199, *p* = 0.029, partial *R*^2^ = 0.021; ADAS13: *β* = 0.186, *T* = 2.984, *p* = 0.003, partial *R*^2^ = 0.037; but not ADNI-MEM [*p* > 0.05]), MCI (MMSE: *β* =  − 0.192, *T* =  − 3.391, *p* < 0.001, partial *R*^2^ = 0.091; ADAS13: *β* = 0.207, *T* = 2.274, *p* = 0.025, partial *R*^2^ = 0.044; ADNI-MEM: *β* =  − 0.112, *T* =  − 2.028, *p* = 0.045, partial *R*^2^ = 0.035), and dementia (ADNI-MEM: *β* =  − 0.430, *T* =  − 2.871, *p* = 0.008, partial *R*^2^ = 0.227; but not MMSE and ADAS [*p* > 0.05]). In contrast, no significant associations were found between amyloid-PET and cognitive decline when tested stratified by diagnostic group. Exploratorily repeating the above described models for a single non-composite memory score (i.e., ADAS-Cog word recognition) revealed congruent results. Further stratifying the analyses by amyloid-status revealed pronounced effects in the Aβ^+^ group (Supplementary Tables 1 and 2). Together, our findings show that tau-PET is a suitable single-modality-based predictor for future cognitive impairment.

### Advanced Braak-stage is associated with faster cognitive decline

**Fig. 3:**
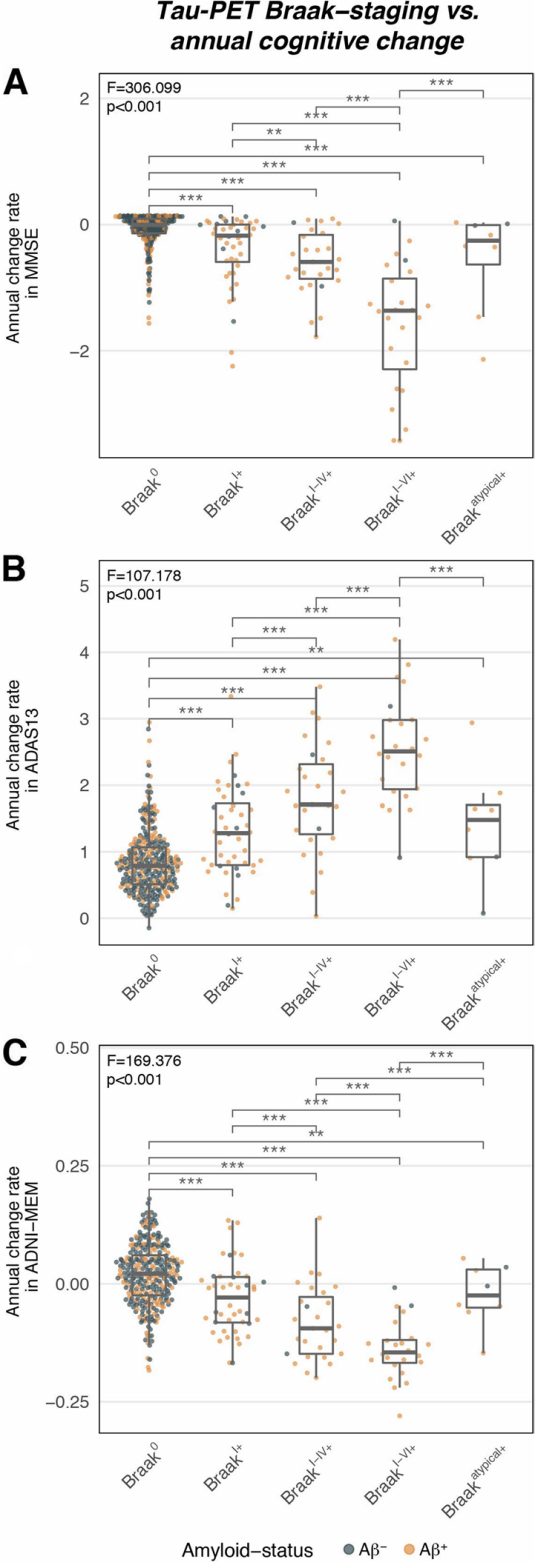
Tau-PET-based Braak-staging versus annual cognitive change rates for the Mini-Mental State Examination (MMSE; **A**), the Alzheimer’s Disease Assessment Scale Cognition 13-item scale (ADAS13; **B**), and the ADNI-MEM score (**C**). Statistics were derived from ANCOVA models controlling for age, sex, education, clinical diagnosis, global amyloid-PET (Centiloid), and the baseline score of the respective cognitive test. Post hoc Tukey tests were used in order to determine differences in cognitive changes between Braak-stage groups; ** = *p* < 0.01, *** = *p* < 0.001

Next, we tested whether more advanced Braak-stage at baseline was associated with faster subsequent cognitive decline using ANCOVAs. We found the expected association between more advanced Braak-stage and faster subsequent cognitive decline consistently for MMSE (F[4,384] = 306.099, *p* < 0.001, Fig. 3A), ADAS13 (F[4,380] = 107.178, *p* < 0.001, Fig. 3B), and ADNI-MEM (F[4,382] = 169.376, *p* < 0.001, Fig. 3C), controlling for age, sex, education, diagnosis, global amyloid-PET, and the baseline score of the respective test. Post hoc Tukey tests confirmed significant differences between sequential Braak-stage groups (all *p* < 0.05). In brief, Braak^0^ subjects showed slowest annual cognitive changes, whereas rates of cognitive decline gradually increased across advancing Braak-stage, with fastest change in Braak^I–VI+^ individuals (Fig. 3). Braak-stage-specific annual cognitive change rates for each cognitive test are summarized in Table 2. Together, advanced Braak-stage increases the likelihood for future cognitive decline. Standardized differences (Cohen’s *d*) in cognitive decline between Braak-stage groups are shown in Supplementary Table 3. Again, this finding supports the view that tau-PET is a promising single marker for prognostication of cognitive decline.

**Table 2:** Annual changes in cognition across Braak-stages

	**Braak**^**0**^	**Braak**^**I+**^	**Braak**^**I–IV+**^	**Braak**^**I–VI+**^	**Braak**^**atypical+**^	***p* value**
MMSE	− 0.08 (0.27)^b, c, d, e^	− 0.39 (0.56)^a, d^	− 0.59 (0.51)^a, d^	− 1.60 (1.01)^a, b, c, e^	− 0.55 (0.80)^a, d^	< 0.001
ADAS13	0.85 (0.48)^b, c, d, e^	1.30 (0.67)^a, c, d^	1.80 (0.83)^a, b, d^	2.55 (0.78)^a, b, c, e^	1.42 (0.84)^a, d^	< 0.001
ADNI-MEM	0.02 (0.06)^b, c, d^	− 0.03 (0.07)^a, c, d^	− 0.08 (0.08)^a, b, d^	− 0.14 (0.06)^a, b, c, e^	− 0.02 (0.07)^d^	< 0.001

Values are presented as mean (SD). *p* values were derived from ANOVAs with post hoc Tukey tests

*MMSE* Mini-Mental State Examination, *ADAS13* Alzheimer’s disease assessment scale, cognitive subscale, *ADNI-MEM* episodic memory composite score

Mean values significantly (*p* < 0.05) different from ^a^Braak^0^, ^b^Braak^I+^, ^c^Braak^I–IV+^, ^d^Braak^I–VI+^, ^e^Braak^atypical+^

To test whether the observed associations between Braak-stage and cognitive decline are consistent across diagnostic groups, the above described analyses were repeated stratified by diagnostic group (i.e., CN, MCI, dementia), controlling for age, sex, education, global amyloid-PET, and baseline cognition. Again, associations between advanced Braak-stage and faster subsequent cognitive decline were observed for CN (MMSE: F[4,229] = 8.308, *p* < 0.001; ADAS: F[4,227] = 11.670, *p* < 0.001; ADNI-MEM: F[4,227] = 8.507, *p* < 0.001), MCI (MMSE: F[4,112] = 46.253, *p* < 0.001; ADAS13: F[4,110] = 16.884, *p* < 0.001; ADNI-MEM: F[4,112] = 36.690, *p* < 0.001), and dementia (MMSE: F[4, 25] = 13.993, *p* < 0.001; ADAS: F[4, 25] = 3.884, *p* = 0.014; ADNI-MEM: F[4, 25] = 9.158, *p* < 0.001). Congruent effects were obtained when tested in Aβ^+^ only (i.e., Supplementary Tables 4 and 5, Supplementary Figure 1), or when repeating the above described analyses using non-parametric Kruskal–Wallis tests which are more robust at smaller sample sizes (all *p* < 0.05). This supports the view that advancing Braak-stage is associated with increased risk for future cognitive decline consistently across diagnostic groups, which supports the use of tau-PET in clinical settings as a general predictive marker for cognitive decline.

### Advanced Braak-stage is associated with higher conversion risk

**Fig. 4:**
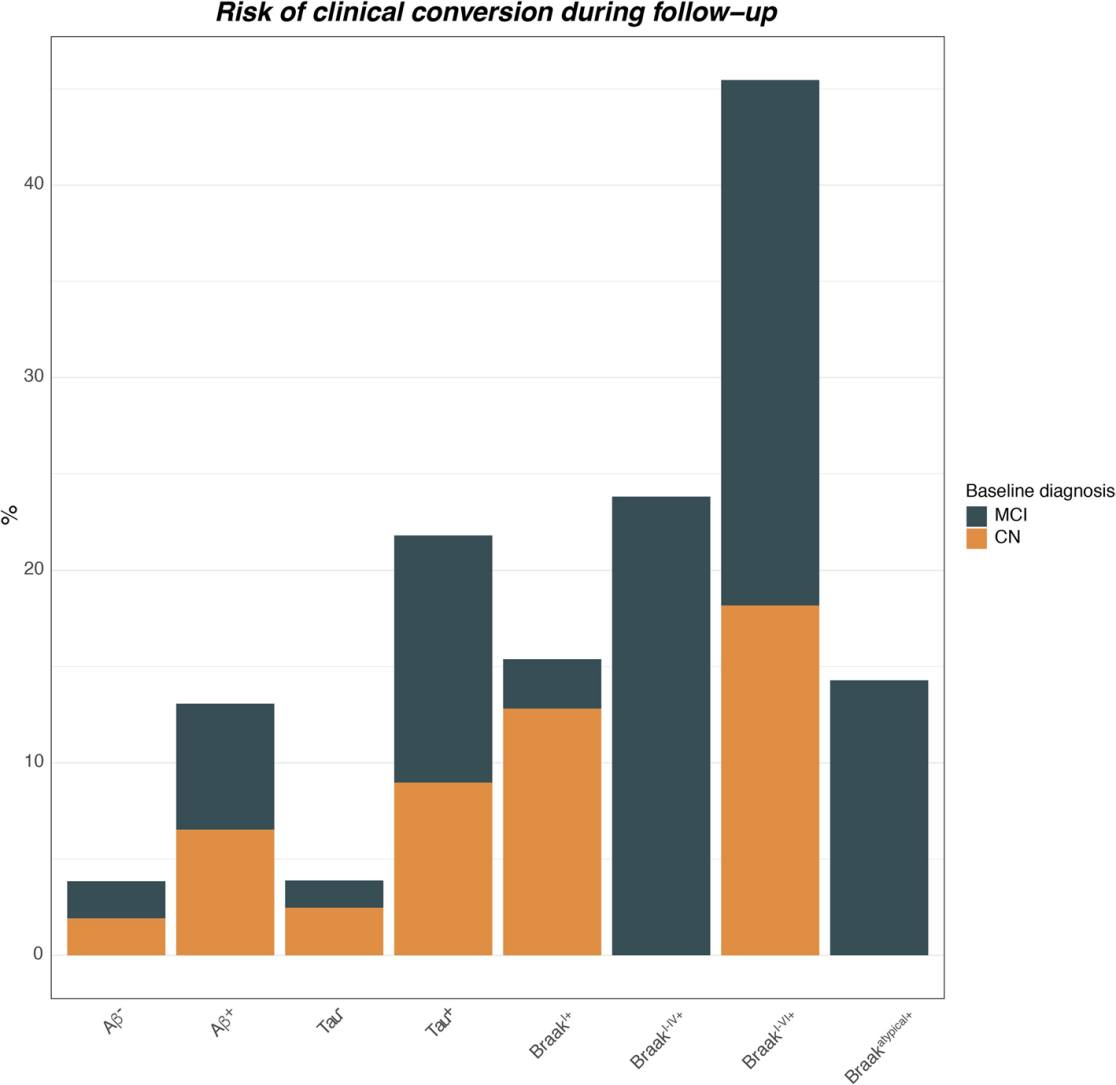
Rates of clinical conversion during follow-up stratified by amyloid-PET positivity, tau-PET positivity and Braak-stage group. Barplots show relative risk of clinical conversion from cognitive normal (CN) to mild cognitive impairment (MCI) or dementia, and from MCI to dementia. Note that subjects with a baseline diagnosis of dementia were excluded from this analysis, since no further diagnostic change can be observed in these participants

Lastly, we compared the predictive accuracy of baseline tau-status and amyloid-status (i.e., global and Braak-stage specific) for future conversion risk from CN to MCI/dementia and from MCI to dementia during follow-up. Note, that 35 participants with baseline dementia diagnosis were excluded from this analysis, since no further diagnostic conversion can be observed in this group. Examining the association between baseline Aβ-status or tau-status and clinical conversion during follow-up, Pearson’s *χ*^2^ test revealed a significant difference between amyloid- and tau-PET positivity and clinical conversion (Aβ-status: *χ*^2^ [1, *N* = 361] = 9.24, *p* = 0.002; tau-status: *χ*^2^ [1, *N* = 361] = 24.962, *p* < 0.001). *χ*^2^ scores were higher for tau-status compared to Aβ-status, suggesting that tau positivity is more critical for conversion than Aβ positivity. Specifically, Aβ^**+**^ individuals had a conversion risk of 13.07% (*n* = 20/153) vs. 3.85% (*n* = 8/208) in Aβ^**−**^ individuals. In contrast, tau^**+**^ individuals had 21.80% (*n* = 17/78) conversion risk, vs. 3.89% (*n* = 11/283) in tau^**−**^ individuals. Thus, tau positivity is associated with a higher risk for future conversion than Aβ positivity alone. Further supporting this notion, we observed a significant association between Braak-stage (Braak^0^ included) and clinical conversion (*χ*^2^ [4, *N* = 361] = 38.925, *p* < 0.001), with a gradual increase in conversion risk across advancing Braak-stage. Specifically, conversion risks were 15.38% (*n* = 6/39) for Braak^I+^, 23.81% (*n* = 5/21) for Braak^I–IV+^, and 45.45% (*n* = 5/11) for Braak^I–VI+^. Individuals classified as Braak^atypical+^ had a conversion risk of 14.29% (*n* = 1/7) (Fig. 4). The results remained consistent when using alternative pre-established CL cut-offs of 30 [31] or 19 [32] instead of tracer-specific SUVR cut-offs for amyloid-PET. Again, these findings illustrate that spatial expansion of tau pathology is strongly associated with the risk of future cognitive decline, while amyloid-PET is prognostically less conclusive, which can be critical for patient-specific disease prognostication.

## Discussion

Here, we systematically investigated tau-PET as a single meaningful predictor of future cognitive decline and clinical AD progression in older adults with and without cognitive impairment. Supporting the clinical usefulness of tau-PET for disease prognostication compared to amyloid-PET, we found that global tau-PET at baseline is a strong predictor of subsequent global cognitive and memory decline across ~ 2 years of cognitive follow-up, clearly outperforming the prognostic utility of amyloid-PET. We further employed PET-based Braak-staging of tau pathology and demonstrate that more advanced Braak-stage is associated with gradually increasing risk for subsequent cognitive decline and clinical conversion to MCI or AD dementia, again clearly outperforming the predictive accuracy of amyloid-PET positivity. Together, our results support the view that tau-PET-based in vivo Braak-staging may be not only diagnostically useful in clinical settings but also provide a clinically powerful approach to estimate patient-specific risk for cognitive decline and clinical AD progression.

In a first step, we show that tau-PET is strongly associated with future cognitive decline and clearly outperforms the prognostic accuracy of amyloid-PET. Importantly, the association between global tau-PET and subsequent cognitive changes remained consistent when additionally controlling for amyloid-PET. In contrast, the association between amyloid-PET and cognition did not reach statistical significance when adding tau-PET to the model. These results support the view that tau pathology is prognostically more conclusive than amyloid, which is in agreement with several previous studies suggesting a close link between tau accumulation and the development of cognitive deficits in AD [3, 4, 6, 33]. A recent longitudinal PET-study in preclinical AD showed that tau accumulation mediates the association between Aβ-burden and cognitive decline [33]. This is congruent with the amyloid cascade hypothesis, suggesting that Aβ is the initial trigger of pathological tau in AD [34, 35] preceding symptom onset by decades [1, 2], while tau is the actual driver of neurodegeneration and cognitive decline [36]. Therefore, a focus on Aβ biomarkers is a key for AD diagnosis, but is likely insufficient for reliable prediction of clinical AD trajectories. When stratifying the analysis across clinical status, associations between more advanced tau-PET and faster cognitive decline were detected across all diagnostic groups while associations between amyloid-PET and cognitive decline did not reach statistical significance. These findings critically extend previous research by showing that tau-PET is a universal and generalizable predictor for future cognitive decline independent of Aβ-burden and clinical status, therefore supporting the clinical use of tau-PET for disease prognostication.

Besides dissimilarities in their temporal proximitity to cognitive decline [1–7], Aβ and tau accumulation show striking differences in their spatial accumulation patterns: While Aβ accumulates rather globally [13], tau spreads in a relatively stereotypical spatio-temporal pattern [37] that is closely associated with clinical status [15]. Therefore, we captured the spatial expansion of tau-PET using a relatively simple and easy approach to assess Braak-staging scheme, where we could confirm that more advanced Braak-stage was associated with gradually accelerated future cognitive decline. Specifically, Braak^0^ individuals (i.e., without evidence of elevated tau pathology) showed slowest annual cognitive changes, whereas Braak^I–VI+^ individuals showed fastest cognitive decline. In line with these findings on continuous rates of cognitive decline, we report that global tau-PET positivity clearly outperformed global amyloid-PET positivity in predicting future clinical conversion risk (i.e., change in diagnosis) during the ~ 2-year follow-up period. Braak-stage specific stratification into risk groups further confirmed gradually increasing conversion risk at more advanced Braak-stages (e.g., 15.38% for Braak^I+^, 23.81% for Braak^I–IV+^, 45.45% for Braak^I–VI+^). While these findings are expected based on previous literature, our results provide numeric risk estimates for Braak-stage specific cognitive decline and conversion risk, which may be clinically useful when it comes to patient-level risk prediction. We caution though that risk-estimates for clinical conversion and cognitive decline are currently based on relatively short follow-up intervals (i.e., the ~ 2-years) and relatively low numbers of conversion events; hence, the findings warrant further validation as soon as larger follow-up data with larger sample sizes for each diagnostic group become available. In line with our results, recent work revealed that more widespread tau pathology was associated with faster future cognitive deterioration [38]. Specifically, tau-PET images were classified into three successive tau stages (negative vs. moderate vs. advanced), using visual interpretation based on the Braak-staging scheme [39]. The advantage of our approach is that we used a standardized and automated procedure rather than visual assessments, which can be easily implemented in clinical PET-analyses workflows and thus minimize inter-rater bias. Together, our findings further support the notion that the spatial expansion of tau pathology holds important information about the risk of future cognitive decline. Our results systematically compare the accuracy of amyloid- vs. tau-PET and Braak-staging as prognostic markers of future cognitive decline across a large cohort of cognitively normal to dementia patients with longitudinal cognitive assessments. We demonstrated that tau-PET is a single powerful marker for the prognosis of future cognitive decline and AD progression which is statistically independent of Aβ and clinical status. Our findings have important implications for clinical trial design in AD, since markers of tau pathology using Braak-staging could be critical in addition to Aβ-markers for matching progression risk among placebo vs. verum groups. In addition, clinical decision making would benefit from a single and accurate predictor of future progression, which could facilitate patient-specific care.

Besides biomarkers of Aβ and tau accumulation, there are numerous other factors that have been associated with cognitive decline and conversion risk, including lifestyle and reserve-related factors (e.g., physical activity, education) [40], genetic risk (e.g. APOE4, BIN1, BDNF) [41–43], neuroimmune markers (e.g., sTREM2) [44], and neurodegeneration [45] as for instance measured with MRI, FDG-PET, or neurofilament light. Thus, a combination of lifestyle, genetics as well as clinical and multi-modal biomarkers is likely to yield higher accuracy for predicting cognitive decline and AD progression. Supporting this, we reported previously that a machine-learning model combining structural MRI, FDG-PET, amyloid-PET, and fluid biomarkers for predicting cognitive decline in AD patients outperformed models based on single modalities/biomarkers [46]. However, multi-modal prediction models including multiple PET scans and fluid biomarkers can be complex and require the assessment of multi-level data which can be challenging to acquire in clinical settings. Also, unspecific markers such as neurodegeneration are often agnostic to the underlying disease process and may be associated with different progression levels depending on the underlying pathophysiology. Thus, our findings on ^18^F-Flortaucipir tau-PET as a single, AD-specific and highly predictive biomarker for cognitive decline and clinical progression are potentially of high clinical use.

Several caveats should be considered when interpreting our results. First, ^18^F-Flortaucipir shows considerable off-target binding in the hippocampus and basal ganglia, which may confound the assessment of tau pathology [47]. Therefore, we excluded regions which are known to be affected by off-target binding. However, influences of unspecific binding remain possible, hence our findings await further replication once sufficient data with second-generation tau-PET tracers (i.e., with a better off-target binding profile) are available. Second, we classified PET using pre-established cut-offs, which is of high clinical use but arbitrarily binarizes a continuous biological process (i.e., Aβ or tau accumulation). Although the currently used tau-PET 1.3 SUVR cut-off was selected based on recommendations for tau-PET [28, 32], we exploratory repeated our analyses by slightly altering the tau-PET SUVR thresholds (e.g., ranging between 1.2 and 1.4), revealing a consistent result pattern. However, a validation of the applied SUVR cut-off in other cohorts will be essential for future investigations to check whether these pre-defined cut-offs generalize across diverse samples and populations. In addition, PET cut-offs may be replaced in the future by more advanced methods such as gaussian-mixed model-based transformation of tau-PET SUVRs to tau positivity probabilities [22, 48]. A third limitation relates to the individual spatial variability in tau deposition patterns. Previous work found that the spreading patterns of tau pathology can be spatially heterogeneous across individual patients. To address this, we applied a relatively simple tau staging scheme (i.e., Braak^I+^, Braak^I–IV+^, Braak^I–VI+^, Braak^atypical^) that does not take into account asymmetry in tau deposition [4] or fine-grained regional differences in the distribution of tau pathology. Only ~ 8% of subjects deviated from this staging-scheme, suggesting that the currently employed Braak-staging system is applicable to a majority of AD patients. Still, we caution that this Braak-staging scheme may not be applicable to patients with atypical AD, characterized by heterogeneous and variant-specific (e.g., posterior cortical atrophy, logopenic variant of primary progressive aphasia) [4] tau deposition patterns. Thus, further studies are necessary to determine the predictive accuracy of tau-PET for future cognitive decline in these rare atypical AD cases [4]. Fourth, PET imaging comes with high costs, radioactive burden, and may not be available for each patient or within each country. Therefore, current investigations of plasma tau markers [49] are of high clinical importance for widespread screening for tau pathology. Plasma screening may be used to select subjects eligible for tau-PET and in vivo Braak-staging, which may allow more accurate risk prediction than single plasma-derived tau measures. Finally, the cognitive scales used in the current study partly overlap (i.e., the word recall subtest of the MMSE is also part of the ADNI-MEM composite score). To exclude that results are driven by the usage of overlapping tests, we ran additional models for an alternative memory score (i.e., ADAS-Cog word recognition), which revealed consistent results with our main analysis using ADNI-MEM.

## Conclusions

Together, we show that tau-PET outperforms amyloid-PET in predicting cognitive decline and clinical AD progression, supporting the notion that tau-PET is—in contrast to amyloid-PET—of high clinical usefulness as a single meaningful predictor of future cognitive decline and clinical AD progression [50]. Importantly, we found that regional tau staging allows more fine-grained risk estimation of future cognitive changes, which can be critical to stratify or match risk groups in clinical trials. From a clinical perspective, our findings suggest that in vivo tau-PET-based Braak-staging may be a valuable tool to identify subjects at imminent risk of cognitive decline.

## Supplementary Information

**Additional file 1: Table 1.** Comparison of global amyloid-PET and global tau-PET as predictors of future cognitive decline, stratified by amyloid-status. **Table 2.** Regression model of tau-PET corrected for amyloid-PET as a predictor of future cognitive decline, stratified by amyloid-status. **Table 3.** Effect sizes between Braak-stage groups and cognitive decline. **Table 4.** Distribution of Braak-stage groups, stratified by amyloid-status. **Table 5.** Association between Braak-stage and cognitive decline in amyloid positives. **Figure 1.** Tau-PET-based Braak-staging versus annual cognitive change rates in amyloid positives.

## Data Availability

All data used in this manuscript are publicly available from the ADNI database (adni.loni.usc.edu) upon registration and compliance with the data use agreement. The data that support the findings of this study are available on reasonable request from the corresponding author.

## Abbreviations

Aβ: Beta-amyloid
AD: Alzheimer disease
ADAS13: Measure of global cognition
ADNI: Alzheimer’s Disease Neuroimaging Initiative
ADNI-MEM: Episodic memory composite score
CL: Centiloid
MMSE: Mini-Mental State Examination
SUVR: Standardized uptake value ratio

## References

[1] Fleisher AS, Chen K, Quiroz YT, Jakimovich LJ, Gutierrez Gomez M, Langois CM (2015). Associations between biomarkers and age in the presenilin 1 E280A autosomal dominant Alzheimer disease kindred: a cross-sectional study. JAMA Neurol.

[2] Jack CR, Bennett DA, Blennow K, Carrillo MC, Dunn B, Haeberlein SB (2018). NIA-AA research framework: toward a biological definition of Alzheimer’s disease. Alzheimers Dement.

[3] Wang L, Benzinger TL, Su Y, Christensen J, Friedrichsen K, Aldea P (2016). Evaluation of tau imaging in staging Alzheimer disease and revealing interactions between β-amyloid and tauopathy. JAMA Neurol.

[4] Ossenkoppele R, Schonhaut DR, Schöll M, Lockhart SN, Ayakta N, Baker SL (2016). Tau PET patterns mirror clinical and neuroanatomical variability in Alzheimer’s disease. Brain.

[5] Jack CR, Knopman DS, Jagust WJ, Petersen RC, Weiner MW, Aisen PS (2013). Tracking pathophysiological processes in Alzheimer’s disease: an updated hypothetical model of dynamic biomarkers. Lancet Neurol.

[6] Bennett DA, Schneider JA, Wilson RS, Bienias JL, Arnold SE (2004). Neurofibrillary tangles mediate the association of amyloid load with clinical Alzheimer disease and level of cognitive function. Arch Neurol.

[7] Serrano-Pozo A, Qian J, Muzikansky A, Monsell SE, Montine TJ, Frosch MP (2016). Thal amyloid stages do not significantly impact the correlation between neuropathological change and cognition in the Alzheimer disease continuum. J Neuropathol Exp Neurol.

[8] Brier MR, Gordon B, Friedrichsen K, McCarthy J, Stern A, Christensen J (2016). Tau and Aβ imaging, CSF measures, and cognition in Alzheimer’s disease. Sci Transl Med..

[9] Aschenbrenner AJ, Gordon BA, Benzinger TLS, Morris JC, Hassenstab JJ (2018). Influence of tau PET, amyloid PET, and hippocampal volume on cognition in Alzheimer disease. Neurology.

[10] Chiotis K, Savitcheva I, Poulakis K, Saint-Aubert L, Wall A, Antoni G, et al. [18F]THK5317 imaging as a tool for predicting prospective cognitive decline in Alzheimer’s disease. Mol Psychiatry [Internet]. 2020; Available from: http://www.nature.com/articles/s41380-020-0815-4. [cited 2021 Mar 19]. 10.1038/s41380-020-0815-4 PMC8758479 32616831

[11] Jack CR, Wiste HJ, Weigand SD, Therneau TM, Lowe VJ, Knopman DS (2020). Predicting future rates of tau accumulation on PET. Brain.

[12] Pontecorvo MJ, Devous MD, Kennedy I, Navitsky M, Lu M, Galante N (2019). A multicentre longitudinal study of flortaucipir (18F) in normal ageing, mild cognitive impairment and Alzheimer’s disease dementia. Brain.

[13] Selkoe DJ, Hardy J (2016). The amyloid hypothesis of Alzheimer’s disease at 25 years. EMBO Mol Med.

[14] Braak H, Braak E (1991). Neuropathological stageing of Alzheimer-related changes. Acta Neuropathol.

[15] Schöll M, Lockhart SN, Schonhaut DR, O’Neil JP, Janabi M, Ossenkoppele R (2016). PET imaging of tau deposition in the aging human brain. Neuron.

[16] Folstein MF, Folstein SE, McHugh PR (1975). “Mini-mental state”. A practical method for grading the cognitive state of patients for the clinician. J Psychiatr Res..

[17] Skinner J, Carvalho JO, Potter GG, Thames A, Zelinski E, Crane PK, et al. The Alzheimer’s Disease Assessment Scale-Cognitive-Plus (ADAS-Cog-Plus): an expansion of the ADAS-Cog to improve responsiveness in MCI. Brain Imaging Behav. 2012;6. Available from: https://www.ncbi.nlm.nih.gov/pmc/articles/PMC3873823/ [cited 2020 Dec 7]. 10.1007/s11682-012-9166-3 PMC3873823 22614326

[18] Crane PK, Carle A, Gibbons LE, Insel P, Mackin RS, Gross A (2012). Development and assessment of a composite score for memory in the Alzheimer’s Disease Neuroimaging Initiative (ADNI). Brain Imaging Behav.

[19] Franzmeier N, Suárez-Calvet M, Frontzkowski L, Moore A, Hohman TJ, Morenas-Rodriguez E (2020). Higher CSF sTREM2 attenuates ApoE4-related risk for cognitive decline and neurodegeneration. Mol Neurodegener.

[20] Preische O, Schultz SA, Apel A, Kuhle J, Kaeser SA, Barro C (2019). Serum neurofilament dynamics predicts neurodegeneration and clinical progression in presymptomatic Alzheimer’s disease. Nat Med.

[21] Desikan RS, Ségonne F, Fischl B, Quinn BT, Dickerson BC, Blacker D (2006). An automated labeling system for subdividing the human cerebral cortex on MRI scans into gyral based regions of interest. Neuroimage.

[22] Franzmeier N, Dewenter A, Frontzkowski L, Dichgans M, Rubinski A, Neitzel J (2020). Patient-centered connectivity-based prediction of tau pathology spread in Alzheimer’s disease. Sci Adv.

[23] Franzmeier N, Neitzel J, Rubinski A, Smith R, Strandberg O, Ossenkoppele R (2020). Functional brain architecture is associated with the rate of tau accumulation in Alzheimer’s disease. Nat Commun.

[24] Franzmeier N, Rubinski A, Neitzel J, Kim Y, Damm A, Na DL (2019). Functional connectivity associated with tau levels in ageing, Alzheimer’s, and small vessel disease. Brain.

[25] Landau SM, Mintun MA, Joshi AD, Koeppe RA, Petersen RC, Aisen PS (2012). Amyloid deposition, hypometabolism, and longitudinal cognitive decline. Ann Neurol.

[26] Landau SM, Lu M, Joshi AD, Pontecorvo M, Mintun MA, Trojanowski JQ (2013). Comparing PET imaging and CSF measurements of Aβ. Ann Neurol.

[27] Klunk WE, Koeppe RA, Price JC, Benzinger TL, Devous MD, Jagust WJ (2015). The centiloid project: standardizing quantitative amyloid plaque estimation by PET. Alzheimers Dement.

[28] Maass A, Landau S, Baker SL, Horng A, Lockhart SN, La Joie R (2017). Comparison of multiple tau-PET measures as biomarkers in aging and Alzheimer’s disease. Neuroimage.

[29] Lemoine L, Leuzy A, Chiotis K, Rodriguez-Vieitez E, Nordberg A (2018). Tau positron emission tomography imaging in tauopathies: the added hurdle of off-target binding. Alzheimers Dement (Amst).

[30] R Core Team (2020). R: A language and environment for statistical computing.

[31] Salvadó G, Molinuevo JL, Brugulat-Serrat A, Falcon C, Grau-Rivera O, Suárez-Calvet M (2019). Centiloid cut-off values for optimal agreement between PET and CSF core AD biomarkers. Alzheimers Res Ther.

[32] Jack CR, Wiste HJ, Weigand SD, Therneau TM, Lowe VJ, Knopman DS (2017). Defining imaging biomarker cut-points for brain aging and Alzheimer’s disease. Alzheimers Dement.

[33] Hanseeuw BJ, Betensky RA, Jacobs HIL, Schultz AP, Sepulcre J, Becker JA (2019). Association of amyloid and tau with cognition in preclinical alzheimer disease: a longitudinal study. JAMA Neurol.

[34] Hardy J, Selkoe DJ (2002). The amyloid hypothesis of Alzheimer’s disease: progress and problems on the road to therapeutics. Science.

[35] Glenner GG, Wong CW (1984). Alzheimer’s disease and Down’s syndrome: sharing of a unique cerebrovascular amyloid fibril protein. Biochem Biophys Res Commun.

[36] La Joie R, Visani AV, Baker SL, Brown JA, Bourakova V, Cha J (2020). Prospective longitudinal atrophy in Alzheimer’s disease correlates with the intensity and topography of baseline tau-PET. Sci Transl Med.

[37] Braak H, Thal DR, Ghebremedhin E, Del Tredici K (2011). Stages of the pathologic process in Alzheimer disease: age categories from 1 to 100 years. J Neuropathol Exp Neurol.

[38] Lu M, Pontecorvo MJ, Devous MD, Arora AK, Galante N, McGeehan A, et al. Aggregated tau measured by visual interpretation of flortaucipir positron emission tomography and the associated risk of clinical progression of mild cognitive impairment and alzheimer disease: results from 2 phase III clinical trials. JAMA Neurol. 2021; Available from: https://jamanetwork.com/journals/jamaneurology/fullarticle/2775982 [cited 2021 Mar 22]. 10.1001/jamaneurol.2020.5505 PMC7885097 33587110

[39] Fleisher AS, Pontecorvo MJ, Devous MD, Lu M, Arora AK, Truocchio SP, et al. Positron emission tomography imaging with [18F]flortaucipir and postmortem assessment of alzheimer disease neuropathologic changes. JAMA Neurol. 2020; Available from: https://www.ncbi.nlm.nih.gov/pmc/articles/PMC7186920/ [cited 2020 Dec 9]. 10.1001/jamaneurol.2020.0528 PMC7186920 32338734

[40] Stern Y, Arenaza-Urquijo EM, Bartrés-Faz D, Belleville S, Cantilon M, Chetelat G (2020). Whitepaper: defining and investigating cognitive reserve, brain reserve, and brain maintenance. Alzheimers Dement.

[41] Corder EH, Saunders AM, Strittmatter WJ, Schmechel DE, Gaskell PC, Small GW (1993). Gene dose of apolipoprotein E type 4 allele and the risk of Alzheimer’s disease in late onset families. Science.

[42] Franzmeier N, Rubinski A, Neitzel J, Ewers M, Alzheimer’s Disease Neuroimaging Initiative (ADNI) (2019). The BIN1 rs744373 SNP is associated with increased tau-PET levels and impaired memory. Nat Commun.

[43] Franzmeier N, Ren J, Damm A, Monté-Rubio G, Boada M, Ruiz A (2019). The BDNFVal66Met SNP modulates the association between beta-amyloid and hippocampal disconnection in Alzheimer’s disease. Mol Psychiatry.

[44] Ewers M, Franzmeier N, Suárez-Calvet M, Morenas-Rodriguez E, Caballero MAA, Kleinberger G (2019). Increased soluble TREM2 in cerebrospinal fluid is associated with reduced cognitive and clinical decline in Alzheimer’s disease. Sci Transl Med.

[45] Jack CR, Petersen RC, Xu YC, O’Brien PC, Smith GE, Ivnik RJ (1999). Prediction of AD with MRI-based hippocampal volume in mild cognitive impairment. Neurology.

[46] Franzmeier N, Koutsouleris N, Benzinger T, Goate A, Karch CM, Fagan AM (2020). Predicting sporadic Alzheimer’s disease progression via inherited Alzheimer’s disease-informed machine-learning. Alzheimers Dement.

[47] Leuzy A, Chiotis K, Lemoine L, Gillberg P-G, Almkvist O, Rodriguez-Vieitez E (2019). Tau PET imaging in neurodegenerative tauopathies-still a challenge. Mol Psychiatry.

[48] Vogel JW, Iturria-Medina Y, Strandberg OT, Smith R, Levitis E, Evans AC (2020). Spread of pathological tau proteins through communicating neurons in human Alzheimer’s disease. Nat Commun.

[49] Palmqvist S, Janelidze S, Quiroz YT, Zetterberg H, Lopera F, Stomrud E (2020). Discriminative accuracy of plasma phospho-tau217 for alzheimer disease vs other neurodegenerative disorders. JAMA.

[50] Bejanin A, Schonhaut DR, La Joie R, Kramer JH, Baker SL, Sosa N (2017). Tau pathology and neurodegeneration contribute to cognitive impairment in Alzheimer’s disease. Brain.

