# Supplementary material for: Tau-PET and in vivo Braak-staging as prognostic markers of future cognitive decline in cognitively normal to demented individuals
Source: Alzheimers Res Ther. 2021 Aug 12;13:137. doi: 10.1186/s13195-021-00880-x (PMC8361801; doi:10.1186/s13195-021-00880-x)
Supplement: Supplementary file 1 — Additional file 1: Table 1. Comparison of global amyloid-PET and global tau-PET as predictors of future cognitive decline, stratified by amyloid-status. Table 2. Regression model of tau-PET corrected for amyloid-PET as a predictor of future cognitive decline, stratified by amyloid-status. Table 3. Effect sizes between Braak-stage groups and cognitive decline. Table 4. Distribution of Braak-stage groups, stratified by amyloid-status. Table 5. Association between Braak-stage and cognitive decline in amyloid positives. Figure 1. Tau-PET-based Braak-staging versus annual cognitive change rates in amyloid positives. [file 13195_2021_880_MOESM1_ESM.docx]

SUPPLEMENTARY:

**Table 1.** Comparison of global amyloid-PET and global tau-PET as predictors of future cognitive decline, stratified by amyloid-status.

|  | **Global Amyloid-PET** | | | | **Global Tau-PET** | | | |
| --- | --- | --- | --- | --- | --- | --- | --- | --- |
|  | β | T | p | partial R^2^ | β | T | p | partial R^2^ |
| *Aβ- (n=213)* | | | | | | | | |
| MMSE | 0.011 | 0.281 | 0.779 | - | -0.004 | -0.119 | 0.905 | - |
| ADAS13 | 0.024 | 0.395 | 0.693 | - | 0.113 | 2.062 | 0.041# | 0.019 |
| ADNI-MEM | 0.012 | 0.320 | 0.749 | - | -0.029 | -0.812 | 0.418 | - |
| *Aβ+ (n=183)* | | | | | | | | |
| MMSE | -0.033 | -0.985 | 0.326 | - | -0.192 | -5.306 | <0.001* | 0.139 |
| ADAS13 | 0.046 | 0.898 | 0.370 | - | 0.230 | 3.961 | <0.001* | 0.083 |
| ADNI-MEM | -0.043 | -1.260 | 0.209 | - | -0.125 | -3.199 | 0.002* | 0.056 |

The model is corrected for age, sex, education, clinical diagnosis, and the baseline score of the respective cognitive test. Bonferroni correction applied, adjusted alpha level = 0.017; significant p-values are marked with *; uncorrected significant p-values (p<0.05) are marked with #.

**Table 2.** Regression model of tau-PET corrected for amyloid-PET as a predictor of future cognitive decline, stratified by amyloid-status.

|  | **Global tau-PET** | | | |
| --- | --- | --- | --- | --- |
|  | β | T | p | partial R^2^ |
| *Aβ- (n=213)* | | | | |
| MMSE | -0.005 | -0.144 | 0.885 | - |
| ADAS13 | 0.112 | 2.027 | 0.044# | 0.019 |
| ADNI-MEM | -0.030 | -0.843 | 0.400 | - |
| *Aβ+ (n=183)* | | | | |
| MMSE | -0.192 | -5.184 | <0.001* | 0.134 |
| ADAS13 | 0.227 | 3.851 | <0.001* | 0.079 |
| ADNI-MEM | -0.120 | -3.014 | 0.003* | 0.050 |

The model is corrected for age, sex, education, clinical diagnosis, centiloid (CL), and the baseline score of the respective cognitive test. Bonferroni correction applied, adjusted alpha level = 0.017; significant p-values are marked with *; uncorrected significant p-values (p<0.05) are marked with #.

**Table 3**. Effect sizes between Braak-stage groups and cognitive decline.

|  | **MMSE** | **ADAS13** | **ADNI-MEM** |
| --- | --- | --- | --- |
| Braak^0^ vs. Braak^I+^ | -0.95 (large) | 0.88 (large) | -0.76 (medium) |
| Braak^0^ vs. Braak^I-IV+^ | -1.70 (large) | 1.83 (large) | -1.53 (large) |
| Braak^0^ vs. Braak^I-VI+^ | -4.00 (large) | 3.31 (large) | -2.45 (large) |
| Braak^0^ vs. Braak^atypical+^ | -1.58 (large) | 1.14 (large) | -0.65 (medium) |
| Braak^I+^ vs. Braak^I-IV+^ | 0.37 (small) | -0.69 (medium) | 0.67 (medium) |
| Braak^I+^ vs. Braak^I-VI+^ | 1.61 (large) | -1.75 (large) | 1.54 (large) |
| Braak^I+^ vs. Braak^atypical+^ | -0.27 (small) | 0.17 (negligible) | 0.11 (negligible) |
| Braak^I-IV+^ vs. Braak^I-VI+^ | 1.28 (large) | -0.92 (large) | 0.80 (medium) |
| Braak^I-IV+^ vs. Braak^atypical+^ | 0.07 (negligible) | -0.46 (small) | 0.77 (medium) |
| Braak^I-VI+^ vs. Braak^atypical+^ | 1.09 (large) | -1.42 (large) | 1.87 (large) |
| Effect sizes were calculated using Cohen’s d.  MMSE = Mini Mental State Examination; ADAS13 = Alzheimer’s disease assessment scale, cognitive subscale; ADNI-MEM = episodic memory composite score | | | |

**Table 4.** Distribution of Braak-stage groups, stratified by amyloid-status.

|  | **Aβ- (n=213)** | **Aβ+ (n=183)** |
| --- | --- | --- |
|  | n | n |
| Braak^0^ | 197 | 96 |
| Braak^I+^ | 10 | 34 |
| Braak^I-IV+^ | 2 | 25 |
| Braak^I-VI+^ | 2 | 22 |
| Braak^atypical+^ | 2 | 6 |

**Table 5.** Association between Braak-stage and cognitive decline in amyloid positives.

|  | **Braak-stage** | |
| --- | --- | --- |
|  | F statistic | p |
| MMSE | F[4,171]=148.817 | <0.001* |
| ADAS13 | F[4,170]=53.817 | <0.001* |
| ADNI-MEM | F[4,170]=97.339 | <0.001* |

The model is corrected for age, sex, education, clinical diagnosis, centiloid (CL), and the baseline score of the respective cognitive test. Bonferroni correction applied, adjusted alpha level = 0.017; significant p-values are marked with *.


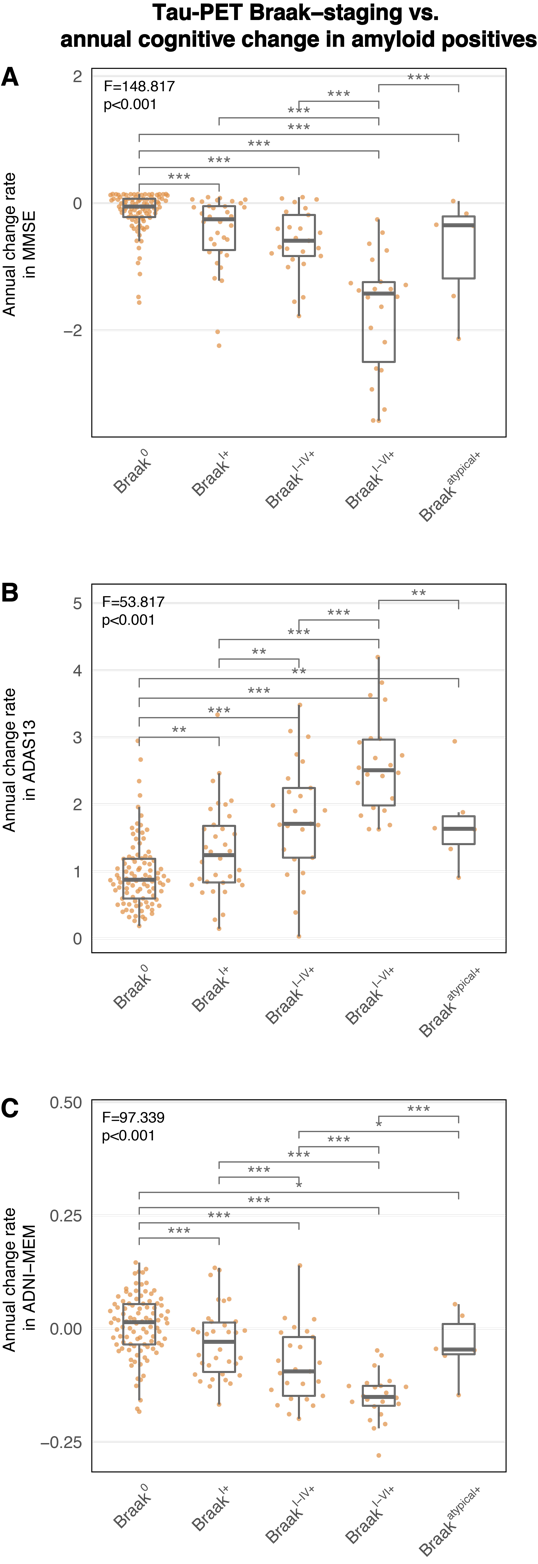


**Figure 1.** Tau-PET-based Braak-staging versus annual cognitive change rates for the Mini Mental State Examination (MMSE; A), the Alzheimer’s Disease Assessment Scale Cognition 13-item scale (ADAS13; B), and the ADNI-MEM score (C) in amyloid positives. Statistics were derived from ANCOVA models controlling for age, sex, education, clinical diagnosis, global amyloid-PET (Centiloid), and the baseline score of the respective cognitive test. Post-Hoc Tukey tests were used in order to determine differences in cognitive changes between Braak-stage groups; *= p<0.05, **= p<0.01, ***=p<0.001.
